# Editorial: Exploring the tumor microenvironment in resectable lung cancer

**DOI:** 10.3389/fonc.2026.1868498

**Published:** 2026-05-12

**Authors:** Alessio Campisi, Federica Pezzuto, Eleonora Faccioli, Andrea Dell’Amore

**Affiliations:** 1 Department of Thoracic Surgery, Thoraxklinik at Heidelberg University Hospital, Heidelberg, Germany; 2 Translational Lung Research Center Heidelberg (TLRC), German Center for Lung Research (DZL), Heidelberg, Germany; 3 Pathology Unit, Department of Cardiac, Thoracic, Vascular Sciences and Public Health (DSCTV), University of Padova, Padova, Italy; 4 Thoracic Surgery Unit, Department of Cardiac, Thoracic, Vascular Sciences and Public Health (DSCTV), University of Padova, Padova, Italy

**Keywords:** immunotherapy, NSCLC, pathology, prognosis, radiomics, stromal biology, tumor microenvironment

In resectable lung cancer, disease behavior is no longer explained by tumor-intrinsic features alone, but increasingly by the immune and stromal context in which the tumor evolves (1,2). Although surgery remains the cornerstone of treatment, growing evidence suggests that outcomes are strongly influenced by the complex interplay between tumor cells, immune components, stromal elements, and systemic host factors (2,3,4). The studies gathered in this Research Topic collectively show that the microenvironment is no longer a biological backdrop, but an active component of clinical interpretation in resectable lung cancer. Taken together, these contributions highlight a shift from a tumor-centered approach toward a microenvironment-integrated model of lung cancer management.

One of the most clinically relevant themes emerging from this Topic is the reinterpretation of pathological response in the era of neoadjuvant immunotherapy. As highlighted by Pezzuto et al., traditional metrics such as major pathological response (MPR) and pathological complete response (pCR), developed in the setting of chemotherapy, are increasingly challenged by immune-mediated patterns of regression that do not fit conventional response frameworks. Lymphocytic infiltration, tertiary lymphoid structures, fibrosis, and granulomatous inflammation can complicate the distinction between residual tumor and treatment-related changes. These observations support the need for refined, immune-adapted pathological criteria, capable of capturing biologically meaningful response beyond simple residual tumor quantification.

A second key message is that prognostic assessment after multimodal treatment should be integrative rather than based on a single-parameter. Qu et al. showed that combining ypT stage, MPR, and nodal pathological response improves prediction of event-free survival compared with single parameters alone, underscoring the clinical relevance of nodal response as a biologically meaningful indicator of treatment sensitivity.

In a different but complementary direction, Bonis et al. confirmed that histologic grade remains a strong predictor of distant recurrence in early-stage lung adenocarcinoma. Although primarily a tumor-intrinsic feature, histologic dedifferentiation likely reflects broader biological processes, including tumor–microenvironment interactions that may favor invasion and metastatic potential.

The stromal compartment also emerges as an active driver of progression rather than a passive scaffold. Chen et al. demonstrated that cancer-associated fibroblasts promote collagen deposition, lymphangiogenesis, and mediastinal lymph node metastasis. These findings support the notion that extracellular matrix remodeling contributes to metastatic dissemination and may represent a potential therapeutic target.

The immune landscape of the TME remains central to understanding both therapeutic opportunity and resistance. Wang et al. provided a comprehensive overview of immune escape mechanisms in lung cancer, including checkpoint signaling, metabolic alterations, and cytokine-mediated immunosuppression, thus offering a framework for interpreting why immunotherapy benefits remain heterogeneous. At a more granular level, a case-based multi-omics study further illustrated how specific tumor subtypes can be associated with highly organized immunosuppressive microenvironments, characterized by stromal–immune interactions that limit effective T-cell infiltration (Wang et al.).

The relevance of the tumor–host interface extends beyond the tissue compartment itself. Tamburini et al. demonstrated that the preoperative neutrophil-to-lymphocyte ratio is independently associated with survival in pulmonary carcinoid patients undergoing surgery, supporting that systemic immune status may capture clinically meaningful aspects of microenvironmental biology.

An especially promising direction is the non-invasive characterization of the tumor microenvironment through imaging. In this context, Ma et al. developed and validated an immune-informed CT-based radiomic signature (CT-RadScore) capable of capturing TME immune phenotypes. Their study demonstrated that radiomics can distinguish immune-active from immune-suppressed tumors, with low CT-RadScore associated with higher immune infiltration and improved response to immunotherapy, whereas high CT-RadScore correlates with proliferative signaling and worse survival outcomes. Moreover, the integration of radiomic features with transcriptomic and single-cell analyses provides a biological basis for imaging-derived biomarkers, linking radiologic heterogeneity to immune activity and tumor proliferation. This contribution is particularly relevant as it bridges imaging, molecular biology, and clinical outcomes, positioning radiomics as a promising non-invasive surrogate of the TME.

Taken together, these studies support a common conclusion: in resectable lung cancer, clinical outcomes are not determined solely by tumor characteristics, but by the interaction between tumor cells and their surrounding microenvironment. Pathological response, recurrence risk, metastatic spread, and therapeutic sensitivity are all shaped by this dynamic ecosystem.

These observations have direct clinical implications. Pathological assessment must evolve to incorporate immune-related features. Prognostic models should integrate tumor, nodal, and microenvironmental variables. Emerging technologies such as radiomics may enable non-invasive and longitudinal assessment of the TME, complementing tissue-based analyses and supporting personalized treatment strategies.

Important challenges remain, including the lack of standardized immune-adapted criteria, the need for prospective validation of imaging biomarkers, and the difficulty of translating microenvironmental complexity into actionable therapeutic strategies.

This Research Topic reinforces a central message: the tumor microenvironment is no longer peripheral to clinical reasoning, but increasingly central for understanding lung cancer biology. Progress in the field depends on integrating surgical, pathological, immunological, and imaging perspectives into a unified and biologically informed framework of care.

Ultimately, the challenge ahead is no longer simply to measure the tumor more precisely, but to understand the disease more comprehensively through the biology of its microenvironment.

